# Architecture and usability of OntoKeeper, an ontology evaluation tool

**DOI:** 10.1186/s12911-019-0859-z

**Published:** 2019-08-08

**Authors:** Muhammad Amith, Frank Manion, Chen Liang, Marcelline Harris, Dennis Wang, Yongqun He, Cui Tao

**Affiliations:** 1 School of Biomedical Informatics, The University of Texas Health Science Center at Houston, 7000 Fannin Street, Suite 600, Houston, 77030 TX USA; 2 Department of Systems, Populations and Leadership, University of Michigan School of Nursing, 426 N. Ingalls St, Ann Arbor, 48109 MI USA; 3 Arnold School of Public Health, University of South Carolina, Columbia, 29208 SC USA; 4 University of Texas, Austin, 78712 TX USA; 5 Center for Computational Medicine & Bioinformatics, University of Michigan Medical School, Room 2017, Palmer Commons 100 Washtenaw Avenue, Ann Arbor, 48109 MI USA

**Keywords:** Biomedical ontologies, Ontology auditing, Quality evaluation, Usability analysis, Knowledge management, Knowledge engineering, Semiotics, Semantic web

## Abstract

**Background:**

The existing community-wide bodies of biomedical ontologies are known to contain quality and content problems. Past research has revealed various errors related to their semantics and logical structure. Automated tools may help to ease the ontology construction, maintenance, assessment and quality assurance processes. However, there are relatively few tools that exist that can provide this support to knowledge engineers.

**Method:**

We introduce OntoKeeper as a web-based tool that can automate quality scoring for ontology developers. We enlisted 5 experienced ontologists to test the tool and then administered the System Usability Scale to measure their assessment.

**Results:**

In this paper, we present usability results from 5 ontologists revealing high system usability of OntoKeeper, and use-cases that demonstrate its capabilities in previous published biomedical ontology research.

**Conclusion:**

To the best of our knowledge, OntoKeeper is the first of a few ontology evaluation tools that can help provide ontology evaluation functionality for knowledge engineers with good usability.

## Background

Ontology evaluation is an important process in the development and maintenance for ontological knowledge-bases, a process that helps ontologists determine if the ontology is of good quality. Literature suggests that readily-accessible and easily usable tooling are needed to assist ontology developers with ontology evaluation task. In this paper, we discuss the design of OntoKeeper, a semiotic-driven ontology evaluation tool. We also discuss results of a usability evaluation by fellow ontologists and their overall assessment of the tool. Furthermore, OntoKeeper’s functionality has been demonstrated in published studies as a tailorable and straightforward method to validate biomedical ontologies. We conclude with future directions that will further improve on this software service.

Ontologies have grown considerably over the last decade. From observation, the Linked Open Data Cloud [1] shows that most of the ontologies online are in the life sciences. However, with all the ontological knowledge bases, there are some considerations — such as maintenance (in terms of updating and upkeep) and quality. Ontological quality entails a variety of issues to verify and validate — logical consistency, veracity of the knowledge, domain coverage, etc.

Based on a sample study of National Center for Biomedical Ontologies (NCBO) BioPortal ontologies, we have shown that most ontologies do not demonstrate documented evidence of evaluation at the time of development [2]. A seminal paper on ontology evaluation by Brank [3] notes that the future of ontology evaluation and quality assessment will lie in automated tools to assist in the process. To the best our knowledge, there are no tools in active use, nor are there standardized methods to evaluate or audit ontologies. We have positioned OntoKeeper, our evaluation software, to facilitate quality evaluation of ontologies and address the gap in evaluation and quality control tools for ontologies.

### Existing tools and research

We have previously noted that many NCBO ontologies do not display documented evidence of evaluation, perhaps indicating lack of validating and verification of the underlying ontological knowledge [4]. This suggests that software tools for ontology evaluation are not readily assessable by the ontology research community, or when available, are not easy to use. To determine what tools and methods are available to the community we queried existing research databases the Association of Computing Machinery (ACM) and Institute of Electrical and Electronics Engineers (IEEE) using “ontology evaluation” or “ontology metrics” for papers published since 2007. The query retrieved 92 unique papers from ACM Digital Library and IEEE Xplore Digital Library. We reviewed the abstract and the body of each paper based on an inclusion criteria of relevancy for automated or semi-automated ontology evaluation software tools. Worth noting, 35 papers discussed methodologies, experimental methods or new metrics to evaluate ontologies. The results of our review after our inclusion criteria yielded six papers that introduced automated or semi-automated tools for ontology evaluation (Table 1).

**Table 1:** Papers surveyed for ontology evaluation software tools

Paper	Method
Ontology Evaluation and Ranking using OntoQA [5]	OntoQA metrics [6]
A Web-Based Ontology Evaluation System [7]	Burton-Jones based; focused on the “subjective” metrics
A Survey on Ontology Evaluation Tools [8]	Survey paper that discussed OntoAnalyser (OntoEdit plugin), OntoGenerator (OntoEdit plugin), WebODE plugin for OntoClean, Ontology Evaluation Tool, and S-OntoEval
Quality Model and Metrics of Ontology for Semantic Descriptions of Web Services [9]	Paper is corrected version [10] that extends the ontology evaluation framework they introduced earlier
An Ontology Selection and Ranking System Based on the Analytic Hierarchy Process [11]	Applies analytic hierarchy process to evaluate ontology through Java-based application tools. Calculates language expressivity, domain coverage, size, consistency, and cohesion
Ranking ontologies in the Ontology Building Competition BOC 2014 [12]	Ranking-based metric system implemented as a web-based tool. Calculates structural, semantic, and term quality

**Ontology evaluation and ranking using OntoQA [**5**]** Tartir and Arpinar introduced OntoQA, which is a metric suite developed for ranking ontologies. The metric suite was implemented through a Java-based web application. The metrics evaluates the ontology on two levels - a “schema” level for structural intrinsic aspect and the instance level for the data from the ontology’s knowledge base. In combination with the metrics and integrated search results from Swoogle, the tool ranks the ontology with respect to the results from Swoogle.

**A web-based ontology evaluation system [**7**]** Jianliang and Xiaowei offered a server-side application that evaluates an ontology using crowdsourcing, with the intent of providing a subjective evaluation of ontologies. Their application tool utilized Burton-Jones and colleagues’ metric suite [13] to provide some subjective measurement of the ontology. In accompaniment with visualization of a concept from the ontology, the crowdsourced user assessed the ontology based on a Likert scale for some of the individual semiotic metrics.

**A survey on ontology evaluation tools [**8] A Survey on Ontology Evaluation Tool by Aruna, et al., was a student paper reviewing a selection of ontology evaluation software tools in conjunction with a set of properties serving as an evaluation criteria - nine properties (two ontology-related and seven related to software performance). The authors evaluated OntoAnalyser, OntoGenerator, OntoClean in WebODE, ONE-T, and S-OntoEval. Four of the tools met their framework criteria for ontology-related properties - assessing syntactic quality (“Language Conformity”) and semantic quality (“Consistency”). Only OntoGenerator was cited in having better software performance.

**Quality model and metrics of ontology for semantic descriptions of web services [**9] Zhu, et al., discussed a web service called ASWebService that supports their own set of ontology metrics grouped into a set of aspects - Content, Presentation, Usage. The evaluation utilized the metrics to compare an ontology with a gold standard ontology to measure each quality attribute from their metrics. The authors’ future direction is to fully automate the evaluation process.

**An ontology selection and ranking system based on the analytic hierarchy process [**11] Groza, et al., introduced a Java desktop application that incorporates Analytical Hierarchical Process (AHP) framework to evaluate and rank ontologies. AHP is a decision hierarchical tree by Thomas Sayat that uses a set of criteria included produce numerical values for specific options [14]. In the context of ontology evaluation, the criteria is a “language expressivity”, “domain coverage”, “size”, “consistency”, and “cohesion”. The resulting numerical values are assigned to a set of examined ontologies. The tool reports the evaluation and domain coverage results.

**Ranking ontologies in the ontology building competition BOC 2014 [**12] Jimborean and Gorza discussed their web-based ontology evaluation and ranking system. The tool incorporated a metric suite that was inspired from OntoQA and various ontology ranking approaches like AktiveRank [15] and OS_Rank [16]. The web-based tool was developed in Java and utilizes OWL-API [17] to extract data from the ontology and Apache Jena [18] for SPARQL querying. The latter was used to support competency questions. Users can attain a ranking score and metric score by simply uploading the ontology and selecting various metrics.

Out of the six papers, none of the tools are available for reuse or public consumption, mainly due to their experimental nature. We also have examined, for each evaluation tool, how metrics are used to measure the quality of the ontology. We assume that metrics provide a means to automate and communicate quality.

While experimental tools have been proposed over the last 10 years, none of them are available in the form of a distributable application or are hosted on a publicly available platform that can promote widespread evaluation. With the availability of widespread and documented evaluation, the ontology community can produce validated and verified ontological knowledge bases with little errors or inconsistencies. OntoKeeper is intended to produce a tool that is available for broad usage for any domain. Most of our work is in the biomedical sphere, but we foresee OntoKeeper being applicable for a wide-range of domains other than biomedicine. Also, OntoKeeper utilizes a metric suite that is easy to use and easy to interpret, so that any ontologist could make the necessary improvements for their ontology. The following sections will describe the background theory behind the metric suite.

Our review of the papers revealed some important observations. We realized how evaluation tools for ontologies were harnessing a set of metrics that were developed by the authors or adapted some pre-existing metrics that has been published, as this would help to quantify the evaluation that can be quickly calculated by a machine. Unique among the other tools was Jimborean and Gorza [12] incorporating some support for competency questions which is sometimes used as a method to evaluate an ontology [19]. Ranking ontologies was a common theme among the papers as it would serve as a benchmark to decipher metric scoring. Of interest, Burton-Jones and colleagues’ semiotic metric was mentioned in two of the six papers. Also, one of the tools mentioned in [8] relied on semiotic theory for ontology evaluation. In the following section, we recall our previous works where we used semiotic metrics [13] to evaluate biomedical ontologies and how these use-cases informed the design of our own software tool, OntoKeeper.

### Ontologies and semiotics

Ontologies are “a formal, explicit specification of a shared conceptualization” [20]. As electronic artifacts, they represent concepts and the semantic relationships that connect them to imbue meaning, context, and reasoning for machines to consume and process. For machines to consume and process ontologies a machine-readable syntax, such as RDF (Resource Description Framework) [21] or OWL2 (Web Ontology Language, v2) [22], are used to serialize the knowledge.

Semiotics, specifically semiotic theory, is the study of the interpretations of signs and symbols [23,24]. Semiotics is organized in three branches–syntactic, semantic, and pragmatic [25]. Building on classic semiotic theory, several authors have recently presented arguments for the application of semiotic theory to contemporary initiatives. For example, Price noted work that demonstrated the value of applying semiotics to understanding information systems and systems analysis, evaluating data model quality, and to evaluating information quality [26]. Applying semiotics theory to contemporary modeling and simulation, Tolk and colleagues defined “syntactical entropy that measures the variety of data representation, semantic entropy that measures the variety of data interpretation, and pragmatic entropy that measures the variety of data utilization” [27].

To some researchers, ontologies are also semiotic artifacts [13,28–30]. Echoing Dividino and colleagues, ontologies are symbolic and meaningful representations of a domain space (semantics), constructed in a graph-based format (syntactical). The utility and understanding of the ontology hinges on social, cultural and environmental factors (pragmatics). Because of the semiotic nature of ontologies, it stands to reason that one way to comprehensively evaluate an ontology is to use evaluation standards rooted in semiotic theory. OntoKeeper utilizes and builds upon the Burton-Jones metric suite for semiotic-based ontology evaluation.

### Burton-Jones, et al. semiotic metric suite

Over a decade ago, Burton-Jones, et al. developed a set of metrics that harnessed semiotic theory to grade an ontology quality [13] (See Table 2). The benefits of this suite is that 1) it is independent of domain specificity or ontology language, and 2) it is a simple and thorough evaluation system. The metric suite is composed of four branches - *syntactic*, *semantic*, *pragmatic*, and *social*. The first three are attributed to branches of semiotics while the fourth is a layer introduced by the authors. The *syntactic* score concerns the machine-readability of the ontology artifact, specifically asking if the ontology “can be read” [13]. The *semantic* score assesses the appropriateness of the entities’ labels within the ontology, or if the ontology “can be understood” [13]. The *pragmatic* score pertains to measuring the utility of the ontology, or if the ontology is “useful” [13]. Finally, the social score measures the ontology status among the community of ontologies (i.e. “Can it be trusted?” [13]). For some ontologies, particularly, those that are new or in-development, the *social* score may be neglected. The *overall quality score* is a composite of *syntactic*, *semantic*, *pragmatic*, and *social*. A high overall score generally means better. Later, we will discuss in *Use Cases*, how this *overall quality score* can be compared to an average of the *overall quality score* from a set of ontologies to determine its quality. Furthermore, as we will discuss later (See *Use Cases*), some of the scores may be adjusted by modifying their weights or removing them from the metric suite.

**Table 2:** Constituents of the semiotic metric suite

Metric	Sub-Metric
*syntactic* (*S*)	
	*lawfulness* (*SL*)
	*richness* (*SR*)
*semantic* (*E*)	
	*interpertability* (*EI*)
	*consistency* (*EC*)
	*clarity* (*EA*)
*pragmatic* (*P*)	
	*comprehensiveness* (*PO*)
	*accuracy* (*PU*)
	*relevancy* (*PR*)
*social* (*O*)	
	*authority* (*OT*)
	*history* (*OH*)

Each of the four core metrics are decomposable into several sub-metrics. With *syntactic*, there is *lawfulness* and *richness*. *Lawfulness* measures adherence to syntactic rules of the ontology profile, while *richness* measures the amount of ontology-related features (*ClassAssertions*, *DomainObjectProperties*, etc.). The *semantic* score involves *interpertability*, *consistency*, and *clarity*. Clarity discerns how ambiguous the term labels may be, while consistency calculates the ratio of inconsistent use of terms–for example, using the term “male” as a class and repeating it as a label for an instance. *Interpretability* involves calculating whether the label is meaningful. *Pragmatic* entails *comprehensiveness* (a measure of the ontology’s domain scope), *accuracy* (veracity of the knowledge embedded in the ontology), and *relevancy* (fulfillment of a specific use case). The *social* score consists of an *authority* score, based on the number of links to the subject ontology, and the *history* score, based on access to the ontology. We will discuss the specifics on how to calculate these scores in a subsequent section.

## Method

### OntoKeeper

OntoKeeper is a Java-based web application that analyzes ontology files (.owl or.rdf) using semiotic metrics from Burton-Jones and colleagues. OntoKeeper is the latest upgraded evolution of the author’s previous tool, SEMS [2]. The current version has refined the metric calculation, improves on interface and functionality, and incorporates natural language generation feature, which is harnessed from the Hootation API library [31].

#### Application architecture

**Fig. 1:**
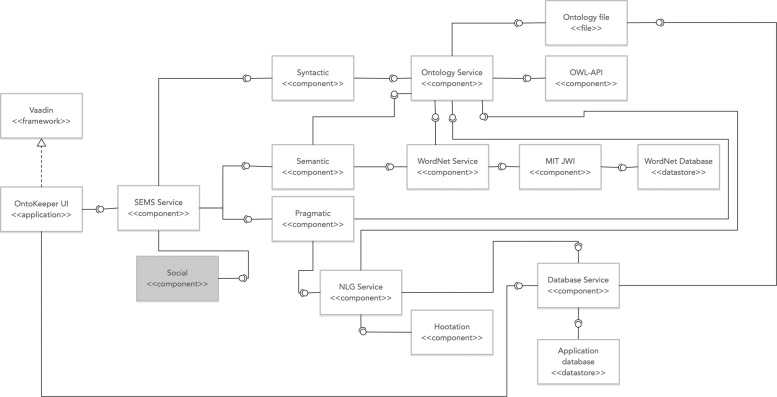
General UML component architecture of OntoKeeper. Grayed components indicate inactive

OntoKeeper was developed with the Vaadin Java web framework (v7.7), along with various third-party API components to provide specific functionality. OntoKeeper also utilized a PostgreSQL database (v9.5.8) to store basic application data and natural language statements. The test version of OntoKeeper was deployed on a Jetty web server (v9), hosted on an Ubuntu v16.04.3 LTS machine (4GB RAM and dual CPU cores). OntoKeeper was primarily developed by one of us (MA) and was evolved from the previous iteration of the tool mentioned in [2]. Figure 1 briefly summarizes the main components, and their interaction with each other.

In the figure (Fig. 1) SEMS Service was a port of the code from the original SEMS web application. It was partitioned by other components (Syntactic, Semantic and Pragmatic components)that were responsible for calculating each of the metrics and sub-metrics, except for the Social component module which is inactive.

Each of the metrics modules heavily relied on the Ontology Service component to parse meta-data and label information from the ontology. The Ontology Service interfaced with either an ontology artifact that has been uploaded (Ontology file) or an ontology that has already previously uploaded and stored in the database through the Database Service. The Ontology Service also required the OWL-API to access functionality for the parsing of an ontology artifact.

In addition, the WordNet Service relied on the Ontology Service to access the label-related information of the ontology. The WordNet Service utilized the MIT JWI, a Java WordNet interface, that queries a WordNet database [32]. WordNet Service primarily provided the word sense information for each token from the labels.

The NLG Service was primarily responsible for the natural language generation of the ontology. It accessed the ontology either through the database (Database Service) or the uploaded ontology (Ontology Service). Also through the Database Service it saved the natural language sentences for each of the triples.

Aside from providing services to other components, the Database Service was also leveraged by the application (OntoKeeper UI component) where the tool stored and retrieved application data to function.

#### Application navigation

Regarding interface design, we aimed to refine the tool to be simple and easy to navigate throughout the various scores, as well as minimizing the amount of information to avoid cognitive overload. The tool was also designed to be responsive to various devices, so in the later part of this section, we present screenshots of the mobile version. The following section describes the interface starting from the login screen to a final screen that showed the final quality score. Also, in this section, we introduce the interface from which external domain experts will access to judge the accuracy of the knowledge embedded in the ontology.

**Fig. 2:**
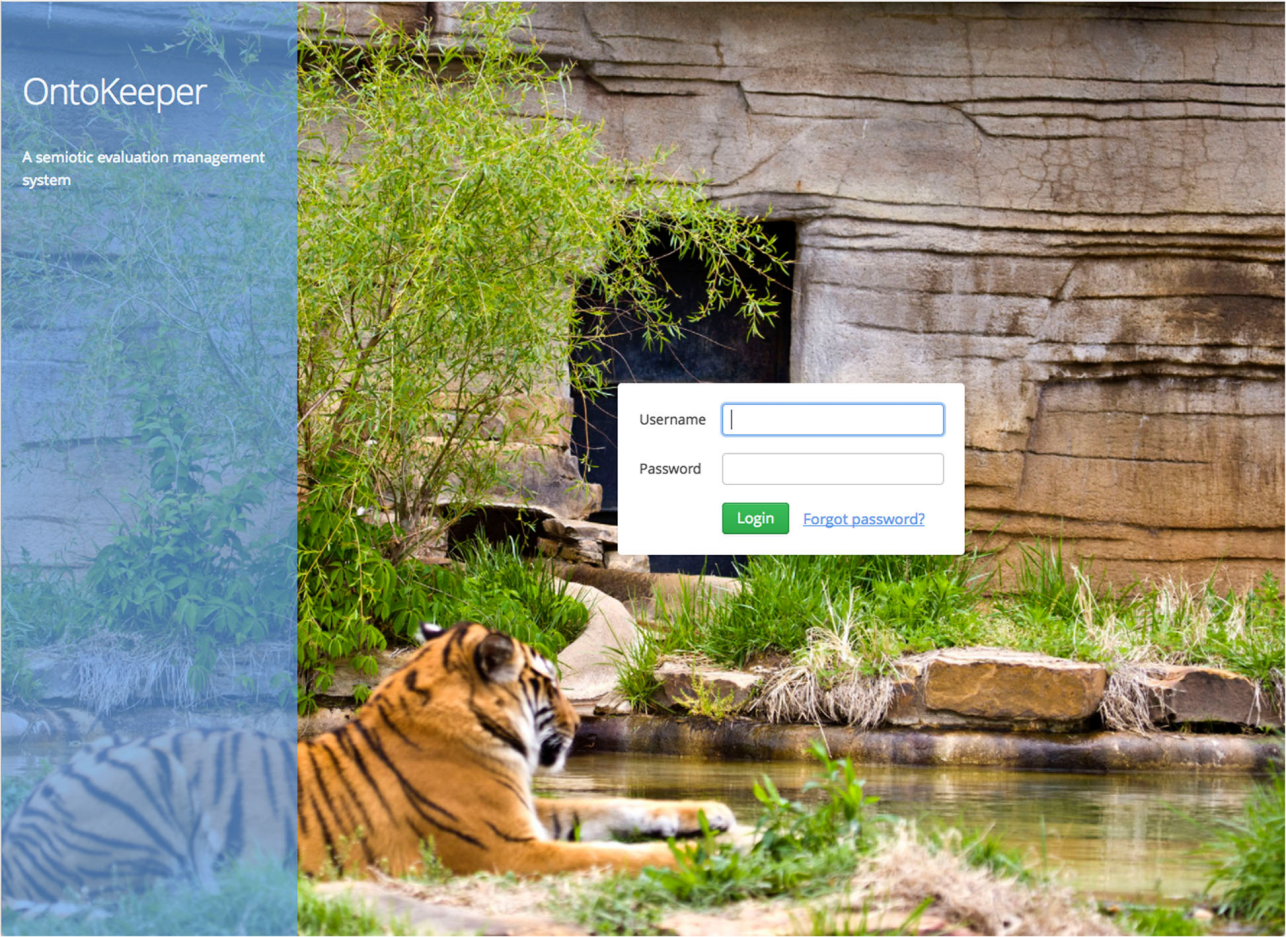
Splash screen

After navigating to the URL address of the application, the ontologist user will encounter the login screen and will be prompted for their username and password (Fig. 2).

**Fig. 3:**
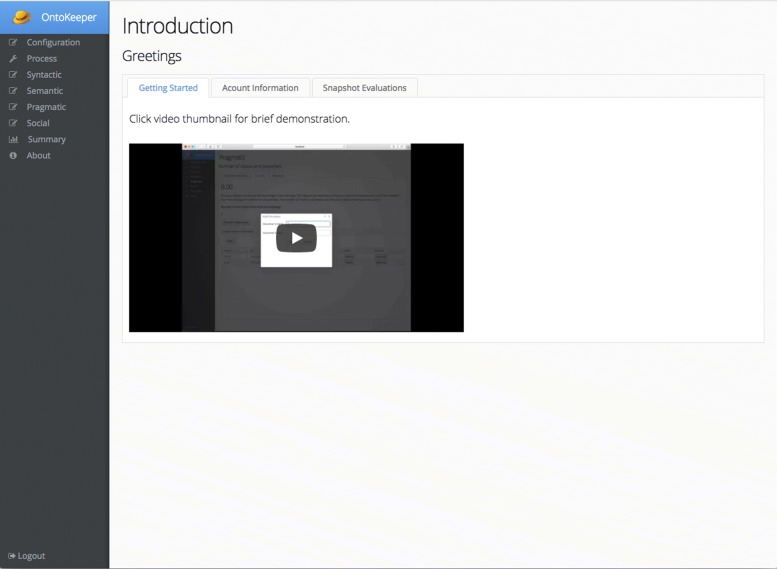
Introduction screen

Figure 3 is the next screen the user views after successfully logging in. The entire application has a visible sidebar menu that allowes the user to navigate between the different sections of the application. The Introduction screen which greets the user after login, has three tabs. The first tab is a short video demonstrating a quick use-case on how to use the tool. The second tab permits the user to change their username or password, and the third tab shows the saved snapshots of scores from previous sessions.

**Fig. 4:**
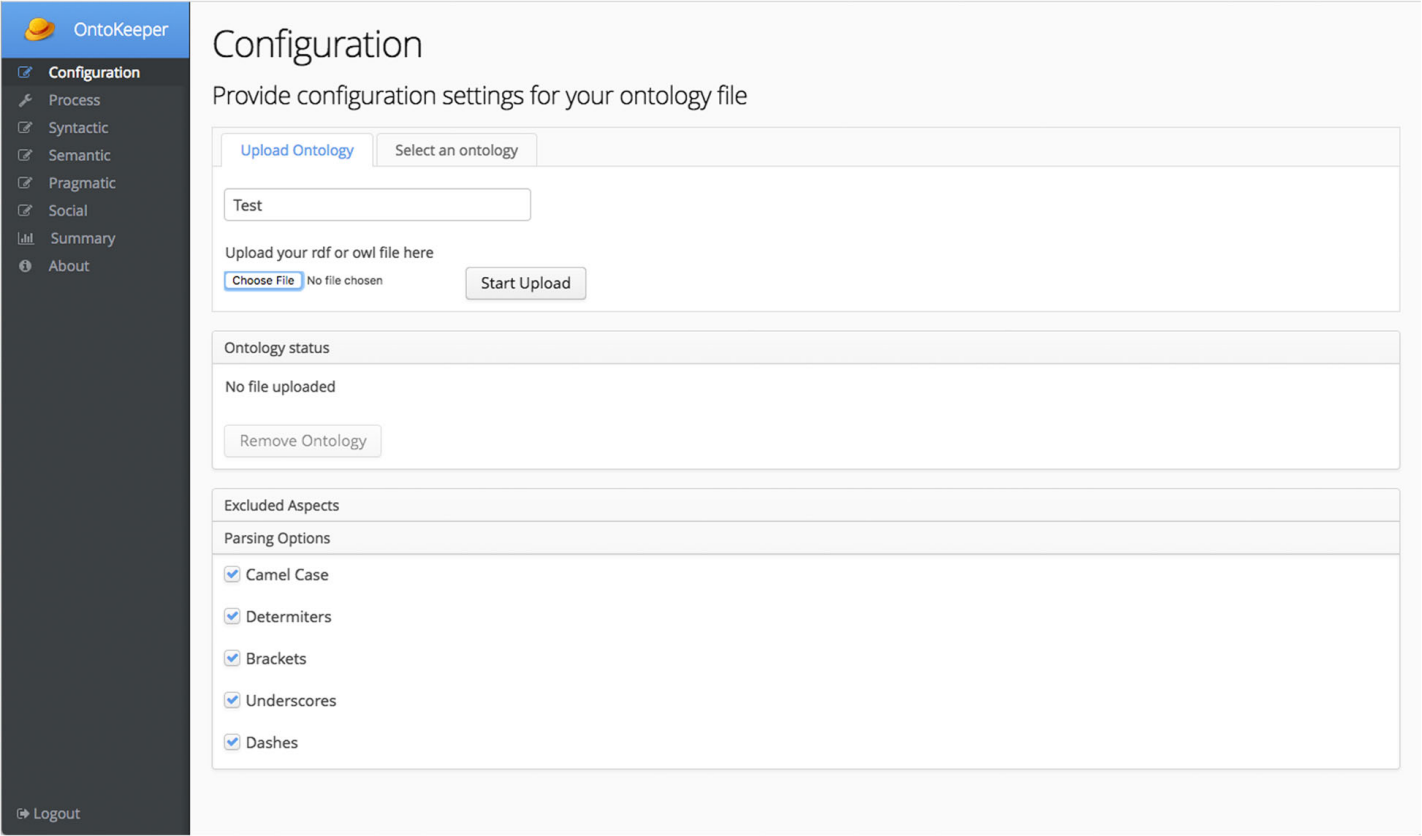
Configuration screen

The Configuration screen (Fig. 4) is where the user starts the process in attaining scores for their ontology or importing an existing ontology the user has uploaded previously. Any ontology file uploaded will be saved into the database automatically for later retrieval. The first panel has two tabs, Upload Ontology and Select an Ontology. The former is where the user will choose the ontology from their machine and upload it to the server. The other tab will present the user with a list of ontologies that the user has previously uploaded. The user can select the ontology and click Import to load the ontology. Currently, we advise the users to merge their ontology (via the Protégé editor) if it imports external ontologies, because the system will calculate the scores based only on what is local to the file and will not follow OWL imports. With a merged ontology, the entities and properties from the imports will be considered into the scoring. In the future, we plan on adding support to automatically import the external ontologies.

The other panels on the Configuration screen includes the Ontology Status panel that indicate whether the ontology has been loaded, with the option to remove the ontology from the session. The Excluded Aspects panel allows the users to exclude scores from the four aspects of *syntactic*, *semantic*, *pragmatic*, and *social*. The Parsing Options panel gives control to users on how to parse non-alphanumeric characters. By default all the options – fixing camel cased labels, removing determiners, brackets, underscores, and dashes – are selected.

**Fig. 5:**
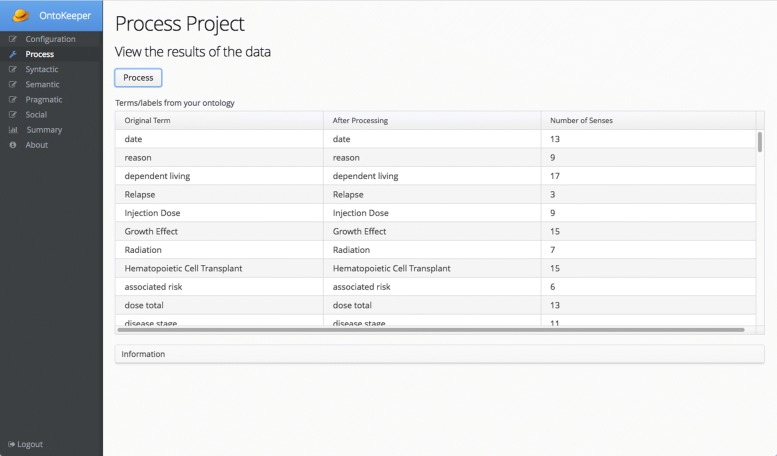
Process screen

After the ontology has been loaded and the session configured, the next screen is the Processing screen (Fig. 5). The labels of the ontology is outputted and displayed in the grid after the Process button has been clicked. The grid displays the original label and the post-processed label based on the configuration. Also, the number of word senses the label has is based on the WordNet database. For labels with multiple tokens, the word senses are added to form an accumulated word sense total.

**Syntactic calculation** The *syntactic* score (Eq. 1) is composed of the *lawfulness* (SL) and *richness* scores (SR). The *lawfulness* is calculated by attaining the total number of axioms (logical and non-logical axioms), which are derived from OWL-API [17]. By instantiating the OWL2DLProfile class with the OWL-API, we also collected the number of violations. Using that count we divide it by the total number of axioms, resulting in the *lawfulness* score.

1 $$  \begin{aligned} S &= w_{s_{1}}*SL + w_{s_{2}}*SR \\ SL &= sl_{v}/AX \\ SR &= sr_{features}/sr_{total features} \\ let\ &AX\ represent\ all\ logical\ and\ non-logical\ axioms \end{aligned}  $$

For *richness*, we used the OWL-API to determine the number of features of the language used in the ontology being evaluated. This was then divided by the number of possible features in the ontology, which for OWL is 39. This quotient provided us with the *richness* score.

**Fig. 6:**
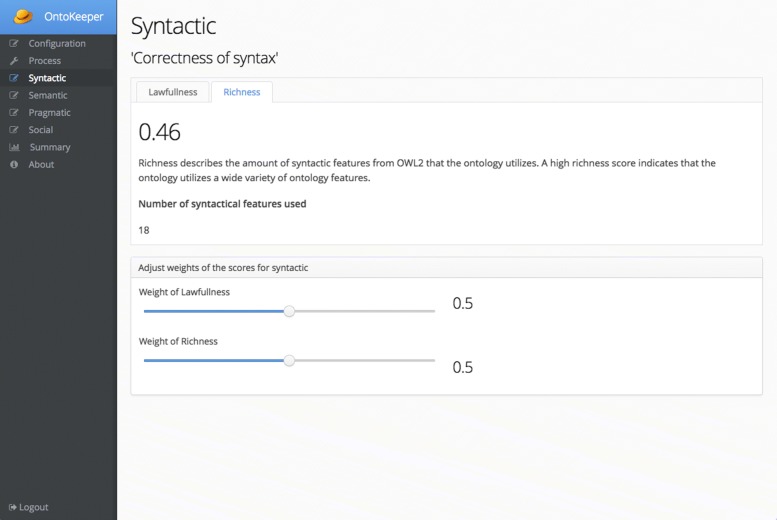
Syntactic screen

Figure 6 shows the Syntactic screen that displays the scores related to the *syntactic* measures. The two tabs relate to the syntactic measures of *lawfulness* and *richness*. Each of these panels displays the scores for these two measures along with a simple explanation of the scores. The other panel contains slider widgets that allow the user to diminish or strengthen one of the scores.

**Semantic calculation**

*Semantic* score (Eq. 2) relies on OWL-API, and WordNet [33] to derive the number of word senses that each word has. For the *interpretability* score (EI), we took the number of unique words from all of the labels that are parsed from the ontology. For each unique word, we used WordNet to discover if the word has at least one word sense, and recorded the total. Using that total, we divided it by the total number of unique words in the ontology. The resulting value is then subtracted from 1 to provide us the *interpretability* score.

2 $$ \begin{aligned} E &= w_{e_{1}}*EI + w_{e_{2}}*EC + w_{e_{3}}*EA \\ EI &= 1-(t_{sense}/t)\\ EA &= 1-\frac{(t_{avg\_senses})}{t}\\ EC &= 1-(d/t)\\ let\ t &= unique\ tokens\ \subset\ ontology\ labels,\\ t_{sense} &= total\ tokens\ with\ one\ word\ sense,\\ t_{senses} &= total\ sense\ per\ token,\\ t_{avg\_sense} &= average\ sense\ per\ token,\\ d &= non-unique\ tokens\ \subset ontology\ labels \end{aligned}  $$

With *clarity* (EA), we utilized the average number of word senses per unique word, and divide that value with the total number of unique words. With that value, we subtracted that from 1 to obtain the *clarity* score.

*Consistency* score (EC) is calculated by counting the number of duplicate words and dividing that figure with the total number of unique words. That value is subtracted from 1 to attain the *consistency* score.

**Fig. 7:**
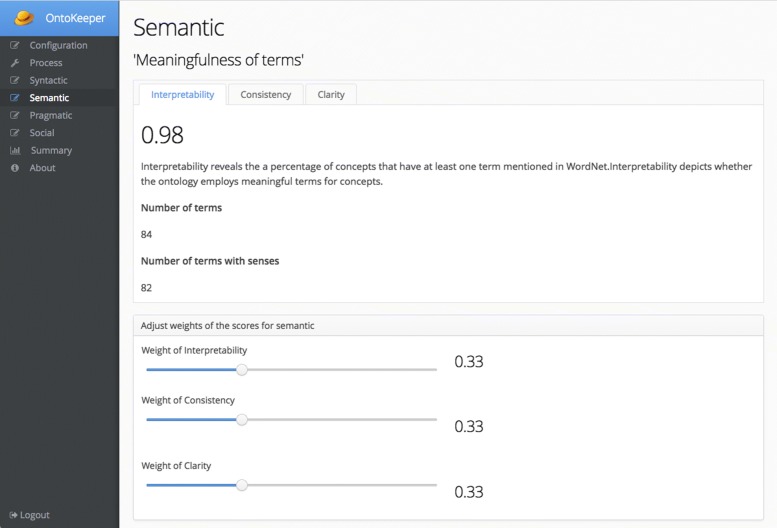
Semantic screen

Similarly, the Semantic screen (Fig. 7) also has the same widget to modulate the three semantic scores of *interpertability*, *consistency*, and *clarity*. There are also three tabs for each of those scores, with an explanation of the scores.

**Pragmatic calculation**

We used the OWL-API to collect the number of classes, instances, data properties, and object properties. The total number of all four of these elements amounted to the total number of elements used to calculate the *comprehensiveness* score (PO) for the *pragmatic* score (P). Also needed was an average number of number of elements (classes, instances, data properties, and object properties) from a group or library of representative ontologies. The total number of elements from the ontology being assessed, was divided by the aforementioned average number of elements. For example, if we have a food-related ontology, we would require the average of classes, instances, and properties from similar food-related ontologies that are available, or we could attain the average from a general ontology repository/library, like NCBO BioPortal, if there is a scarcity of similar ontologies. All in all, the numeric values results in the *comprehensiveness* score of the ontology.

The *accuracy* score (PU) relies on the Hootation API (See “Hootation” section) and external experts. All of the logical axioms from the ontology were translated into natural language. The external domain experts assessed if each statement was true or false. The number of true statements was collected and averaged, and the final value produced the *accuracy* score.

3 $$ {\begin{aligned} P &= w_{p_{1}}*PO + w_{p_{2}}*PU + w_{p_{3}}*PR\\ PO &= CIDO_{n}/CIDO_{average}\\ PU &= AX_{true_{n}}/AX_{logical_{n}}\\ let\ CIDO_{n} &= \{ classes_{n} \cup instances_{n} \cup data\ properties_{n}\\ &\quad\cup object \ properties_{n},\}\\ CIDO_{average} &= average\ from\ set\ or\ library\ of\ ontologies,\\ AX_{logical\_axioms} &\subset\ AX,\ logical\ axioms\ from\ all\ axioms,\\ AX_{human} &\approx AX_{logical\_axiom},\\ natural\ &language\ translation\ of\ axioms,\\ AX_{true_{n}}, &number\ of\ true\ AX_{human},\\ AX_{logical_{n}},\ &number\ of\ AX_{logical\_axioms} \end{aligned}}  $$

*Relevancy* score (PR) is not supported in OntoKeeper, as it a score that is specific to a use-case defined by the evaluator. For example, an evaluator may create a set of competency questions and calculate the percentage of adherence for the questions to determine the *relevancy* score. *Relevancy* is understood as being a score to measure performance of a task, specifically a user-defined task.

**Fig. 8:**
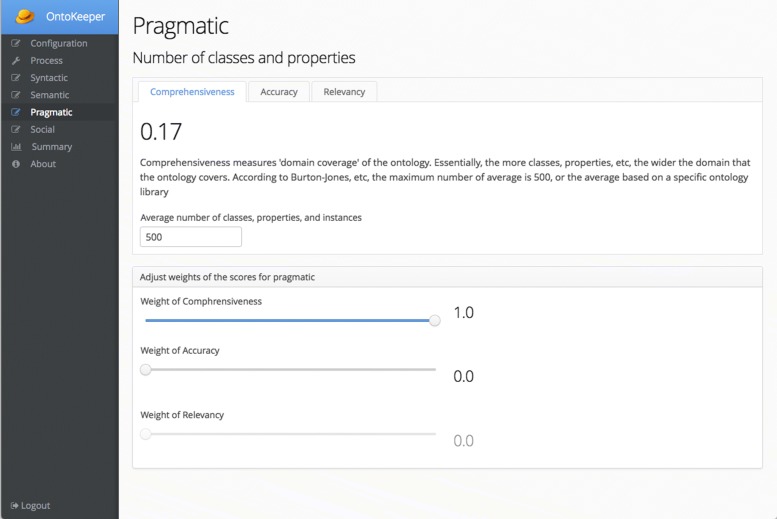
Pragmatic screen

Most of the calculations, are automated, but the *pragmatic* scoring is a bit more involved. Figure 8 shows the Pragmatic screen, and like the previous, it also has slider widgets to control the influence of the *pragmatic* scores. It has three tabs for each of the *pragmatic* scores. The first tab for *comprehensiveness*, displays its score and has a text field for the user to input the average number of ontology elements (classes, properties, and instances). This average value may vary depending on the number of ontologies that are being compared. In our previous study [34], we noted that this number may vary (i.e. 1,277,993 for NCBO Bioportal, 169,862 for a set of drug ontologies). In [13], Burton-Jones, et al. used 500, but over the last decade the size of ontologies have greatly increased, and the *comprehensiveness* score may elicit a value greater than 1. What we have performed, and what is recommended by [13] is to collect a set of ontologies that are of a similar domain and record the total number of elements to input.

**Fig. 9:**
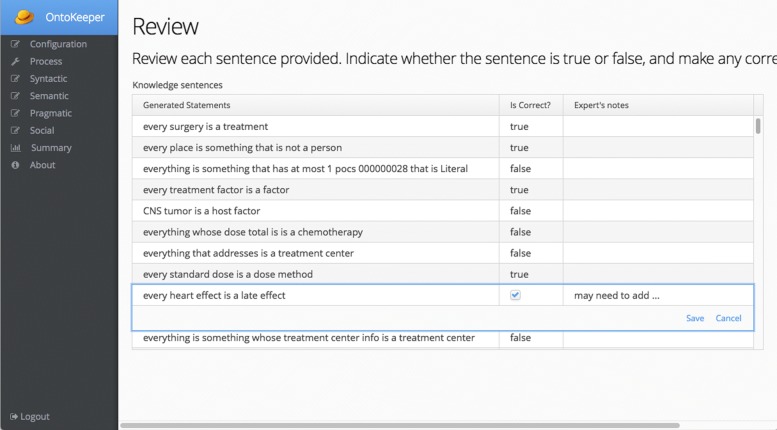
Review of the natural language statements from the axioms

**Fig. 10:**
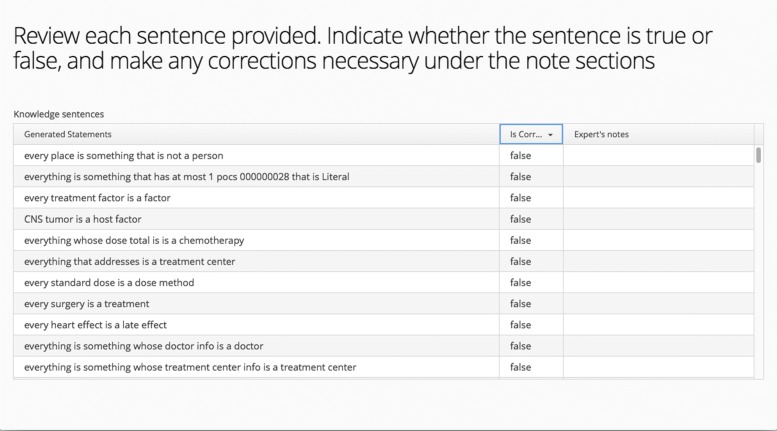
Expert view of the natural language generated statements from an invite link. Same grid UI as Fig. 9

**Fig. 11:**
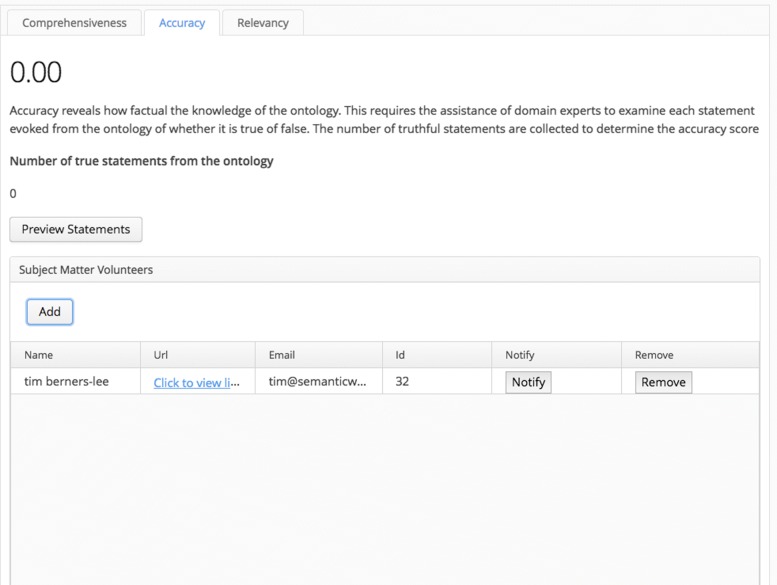
Accuracy tab screen

The second tab for the Pragmatic screen is more involved. Like all the other tabs, it displays information about the sub-score, but it also has functionality to enlist volunteer domain experts to assess the truthfulness of the ontology. The Preview Statements button allows the user to view the list of natural language statements that are from the ontology’s axioms (See Fig. 9). This Review screen will be the same UI as what the enlisted domain experts will experience (See Fig. 10). From Fig. 11, there is also a panel labeled Subject Matter Volunteers. In this widget, the user adds the domain experts to be sent an invitation to examine the user’s ontology. From this panel, the user can remind the volunteers to participate and also view their private link to access their unique grid to review the ontology (Fig. 10). In the review, the volunteer can indicate whether the statement is true or false, and add any notes.

**Hootation**

Hootation API is a Java library based on natural language generation (NLG) components from the Agile Knowledge Engineering and Semantic Web Group’s semantic web application for generating quiz questions [35]. At the time of our past study [31], only 14 logical axiom types were supported, but currently Hootation supports 25 logical axioms types.

A few of the metrics provided through OntoKeeper requires external participants and resources. One such metric (*accuracy*) needs domain experts to assess the veracity of the triples in the ontology. Most domain experts are not familiar with ontology languages or tools. Exporting the logical axioms to human readable language would enable accessibility for domain experts with little ontology experience, even though the knowledge triples are expressed in descriptive logic.

**Social calculation** Also, due to technical limitations, the current iteration of OntoKeeper does not calculate the *social* score (Eq. 3). However, the score is comprised of the *history* score (OH) and *authority* score (OT). The *authority* score is based on an average times that ontology has been accessed within a library of ontologies, and the *history* score is calculated by the number of ontologies of a certain library that links to the ontology.

4 $$ O=w_{o_{1}} * OT + w_{o_{2}} * OH  $$

**Overall Quality Calculation** The *overall quality* (Eq. 4) is a composite score of *semantic* (E), *syntactic* (S), *pragmatic* (P), and *social* score (O). Each score is modulated with weights ($ w_{q_{n}} $) to balance their degree of strength. In a previous publication, we noted how the weights can be leveraged to provide a more accurate composite score among similar ontologies [34].

5 $$ Q=w_{q_{1}}*S + w_{q_{2}}*E + w_{q_{3}}*P + w_{q_{4}}*O  $$

**Fig. 12:**
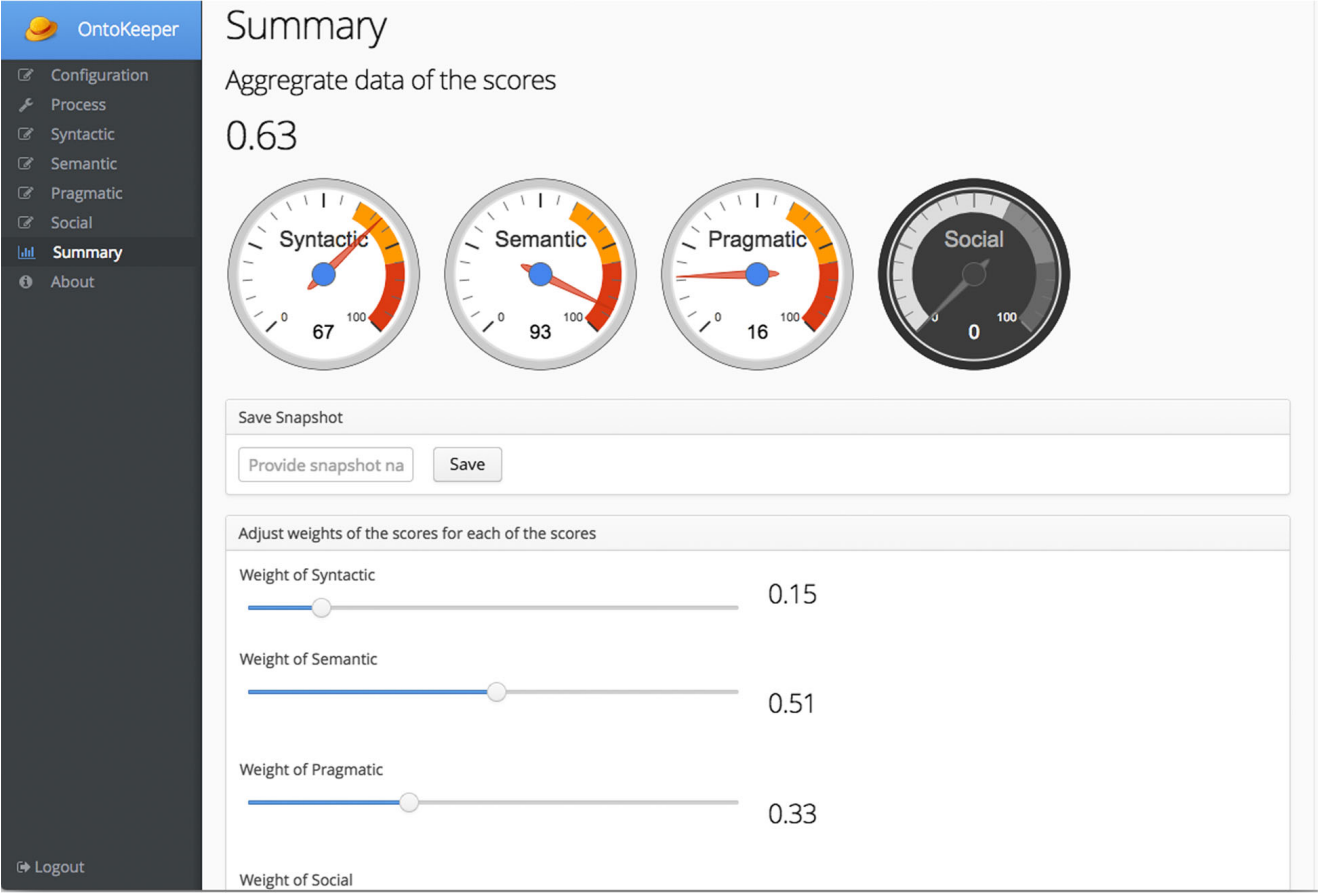
Summary screen

The final screen of importance is the Summary section (Fig. 12). In this screen, the *overall quality* score is displayed along with some visualizations to indicate scores for each of the quality aspects. As noted earlier, *social* score is not supported and thus grayed out on the UI. Similar to the sub-score screens, the user has the option to adjust the strengths of each score. In Fig. 12, for demonstration purposes, the *syntactic* score is weighted at 0.15, *semantic* is weighted at 0.51, and *pragmatic* at 0.33. The final scoring of the session can be saved for archiving using the Save Snapshot panel.

**Fig. 13:**
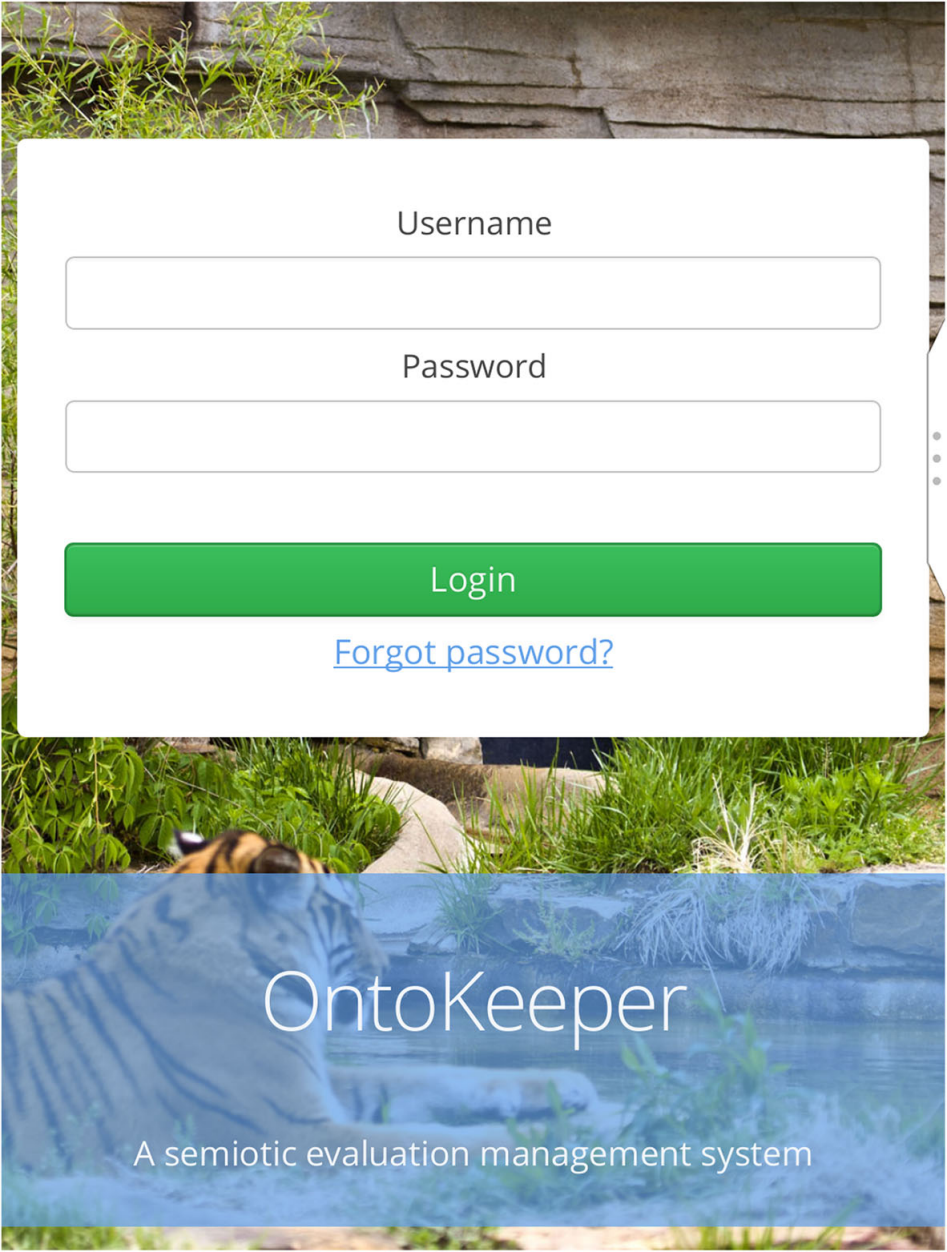
OntoKeeper rendered from smartphone device

**Fig. 14:**
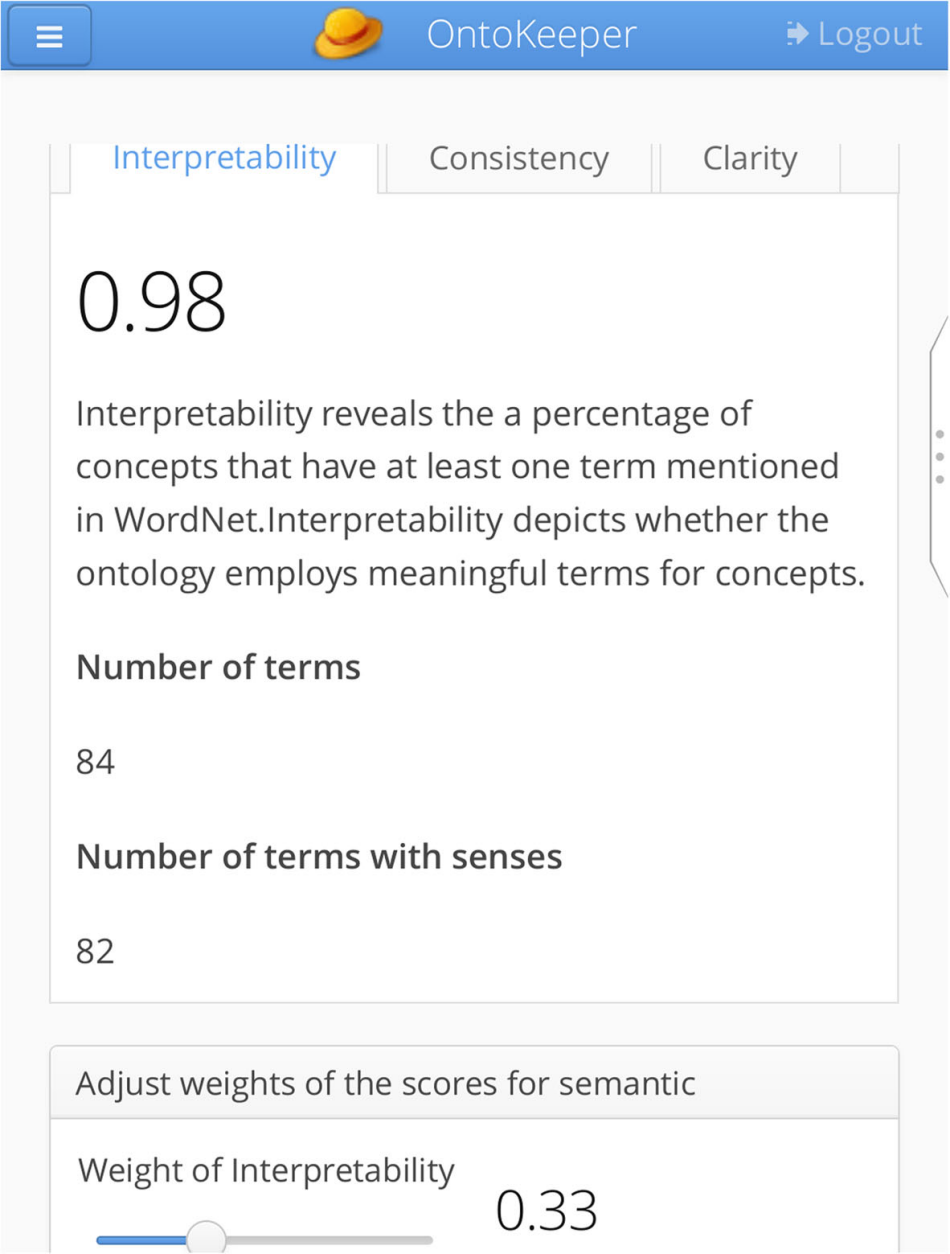
OntoKeeper rendered from smartphone device

Of some worth, the application is also usable through mobile device by way of a responsive design. Figures 13 and 14 show the login screen and the Pragmatic screen rendered from an Android smartphone. With a streamlined interface and adaption to various screen sizes, we foresee that in the future this application could be usable for mobile users. Further refinement of the interface is still needed and the possibility of an ontology artifact residing on someone’s smartphone is remote. For the usability testing, as we will introduce in the next section, our evaluators utilized their desktop instead of their portable devices.

### Usability evaluation

Five of the co-authors (CT, YH, CL, DW, and FM), who have published research and development experience with ontologies, participated in assessing OntoKeeper independently. None of the five co-authors were involved in the development of OntoKeeper. Each participant was furnished with a username and password to login, and each participant did not receive any guidance. Each user were left to their own to operate the tool by uploading an ontology of their choice and explore the tool without any intervention. After reviewing and testing the tool, each participant completed a survey using the System Usability Scale (SUS) [36,37] to appraise the tool. The SUS instrument is a simple 10-item survey using a Likert scale for each item (1=strongly disagreed, 5=strongly agreed). Additionally, SUS is known for its reliability with a small sample [38]. The scores were compiled and discussed in the next section. Lastly, the survey provided free text space to allow further comments that are not covered by the survey.

## Results

### Usability results

For each participant, we collected the results of the System Usability Scale (SUS) survey from all five of the participants and calculated the scores [37] (See Table 3). The average SUS score was 93.5, and participant scores ranged from 87.5 to 100. According to usability studies, the baseline score for average usability using the SUS scale is 68 [38]. With a score in the high 80s and above, it reasonable to assume that participants agreed that OntoKeeper had very high attributes in usability. Usability experts would place the score in the top 96–100 percentile with a grade of “A+” [39].

**Table 3:** *p* notation represent individual participants ratings

SUS Items	*p*_1_	*p*_2_	*p*_3_	*p*_4_	*p*_5_	*μ*(*σ*)
I think that I would like to use this system frequently	4	5	5	5	5	4.8 (0.45)
I found the system unnecessarily complex	1	1	1	1	1	1 (0)
I thought the system was easy to use.	5	5	3	5	5	4.6 (0.89)
I think that I would need the support of a technical person to be able to use this system.	2	5	1	1	1	2 (1.73)
I found the various functions in this system were well integrated.	5	5	5	5	5	5 (0)
I thought there was too much inconsistency in this system.	1	1	1	1	1	1 (0)
I would imagine that most people would learn to use this system very quickly	5	5	5	4	5	4.8 (0.45)
I found the system very cumbersome to use.	1	1	1	1	1	1 (0)
I felt very confident using the system.	5	5	5	5	5	5 (0)
I needed to learn a lot of things before I could get going with this system.	2	1	4	1	1	1.8 (1.30)
**SUS Calculated Score**	**92.5**	**90**	**87.5**	**97.5**	**100**	

Values in parentheses are the standard deviation.Values derived from Likert scale (1=strongly disagreed, 5=strongly agreed)

Also, we calculated the standard deviation for each of the items to verify any divergence of opinion. For eight items, there appeared to be uniformity in opinions based on the standard deviation values. However, for two items there appear to be some variability:

- *I think that I would need the support of a technical person to be able to use this system*.
- *I needed to learn a lot of things before I could get going with this system.*

We deduced that these two items are similar in nature, addressing the need for guidance or simple learning material. Noted earlier, no assistance was given, and participants independently operated the tool on their own volition. The Introduction screen featured a video demo, but we did not ascertain whether the participants watched the video. Although, one user noted that a more detailed video would be helpful.

Each participant had an opportunity to provide feedback (positive, negative) of his or her experience using OntoKeeper. Some of the feedback hinted at suggestions for improvement. Regarding positive feedback, users noted the ease of use of the application and the accessibility of the tool for ontologists of varying expertise.

Users suggested that the *overall quality* score should be persistent throughout the session without having to navigate between tabs. Users also noted challenges with the grid that displays the natural language statements, where there may be numerous translated axioms. This could potentially lead to difficulties for domain experts who are tasked to review the grid of natural language statements. Ideas such as a filter or suggesting an alternative way to display natural language statements might solve this issue.

Some users indicated difficulty comprehending specific parts of the application. One user specified providing more concise instructions on the interface. This issue might harken back to the need for better learning material or an interface with more guidance involved.

### Use cases

We have utilized the semiotic metric suite in two previous studies. In one study, we employed the semiotic-based evaluation system on a set of NCBO ontologies and drug ontologies. The goal was how to effectively use the metric suite to provide meaningful metrics. The other study involved the use of natural language generation, a sub-field of natural language processing, where data is transformed to free text for human understanding of the data. The goal of using natural language generation is to provide better facilities for non-technical domain experts to assess ontologies. These previous works offered were incorporated and consolidated into the OntoKeeper platform to automate the ontology evaluation process for assessing biomedical ontologies in the form of a software application.

#### Utilizing semiotic metrics for drug ontology evaluation

We had utilized this metric suite to evaluate a group of NCBO drug ontologies [34]. In that study we were posed with the question on how to use this metric suite to precisely evaluate a group of drug ontologies. From a random sample of 64 ontologies (from September 2015 among most frequently visited) from the NCBO Bioportal, we calculated each ontology using the metric, and recorded the mean for each of the scores and the *overall quality* score for the sample. This provided us a “gold standard” to evaluate a group of drug ontologies that included RxNORM [40], Veterans Health Administration National Drug File (VANDF) [41], The Drug Ontology (DRON) [42], The Drug-Drug Interactions Ontology (DINTO) [43], Vaccine Ontology (VO) [44], and Pharmacovigilance Ontology (PVOnto) [45].

We also postulated if one were to create a new drug ontology or compare an existing drug ontology with other drug ontologies, that it would be suitable to compare ontologies from a similar domain or sort. We recorded an aggregated score of drug ontologies to create our gold standard comparison but modified the weights based on some observed strengths and weakness of the drug ontologies. The end result yielded modulated weights for each of the scores, specific for drug ontologies.

#### Natural language generation for ontology evaluation

Natural language generation (NLG) offers a feasible method to close the gap between domain experts with no ontology experience and ontologists. NLG is particularly useful for allowing domain experts to assess the veracity of the knowledge that is encoded in an ontology, considering that the coded axioms are translated into natural language free text.

We explored the feasibility of using NLG for the task of knowledge validation using an NLG engine for OWL 2 [31]. We produced natural language statements from logical axioms from three ontologies – “People” ontology [46], Time Event Ontology [47], and Informed Consent Ontology [48]. We had evaluators familiar with each of the ontologies to examine the quality of the natural language output [31]. Overall, the evaluators indicated that most of the output provided clear natural language sentences for each of the triples.

## Discussion

Two items from the survey indicated some issue with learning some of the features of OntoKeeper in order to get started. We may need to devise some alternatives besides the introductory video that could help users better operate the tool quickly - integrated user-generated wiki, guided prompts, etc. While the System Usability Scale has some reliability a with small sample users [38], additional evaluators are needed to provide a significant assessment. Aside from the usability, we also acknowledged some technical limitations. As we discussed before, social score is a feature that is unavailable for a variety of reasons. Specifically we have to determine what are the ontologies that are linking to the subject ontology. However, in light of the difficulty in automating this feature, we could implement some manual input to allow the user to add the number, until a feasible automated solution is available. In addition, we also have to determine the number of times the ontology has been accessed. Similarly, with the comprehensiveness tab, the user has to have a priori knowledge of the number of ontology elements from domain-relevant library of ontologies. Overall, there may be a need to have a stored library or database of ontologies that OntoKeeper can access and gather some of the data needed for the aforementioned scores.

OntoKeeper uses the Hootation API that translates the ontology’s axioms to natural language statements. However, one user noted that the URI (Uniform Resource Identifier) for an entity appeared in a sentence instead of the label. This might be due to an unmerged ontology or some other issue we need to investigate. If it is the former, OntoKeeper may need to have some implementation in place that will import the external ontologies and merge them to the user’s ontology.

Overall, some of these limitations can be surpassed with modifications in the design, further development of the tool, and additional hardware resources. Currently, the tool resides on our development server. Our eventual goal is provide this tool as a service for knowledge engineers to utilize. One idea to extend OntoKeeper is to allow users to publish their scores publicly through OntoKeeper. We envision a public portion of the tool that lists ontologies that have been tested, along with supplementary data like the natural language statements. This might be beneficial for researchers to document the evolution of their ontologies or publically announce the quality of their knowledge-base. Other ideas include having an open API from the server to allow external tools, like Protégé, to leverage OntoKeeper’s backend services. We also alluded to mobile, and we may investigate the feasibility of mobile technologies for ontology evaluation.

## Conclusion

Our review of the literature for automated ontology evaluation tools show the lack of software that could be used by ontologists and researchers to measure the overall quality of ontologies. We presented OntoKeeper as the latest iteration of our contribution towards ontology evaluation and quality assurance. OntoKeeper is a web-based application that permits ontologist users to grade the quality of their ontology based on semiotic measures devised by Burton-Jones and colleagues. The system was designed for public use, and we intend to make this service public and refine specific aspects of the metric suite. With five evaluators, we surmised that the system is deemed to be usable with a SUS score above the baseline. Nonetheless, there is further work needed to enhance the usefulness of the tool for ontologist users.

## Data Availability

Not applicable.

## Abbreviations

ACM: Association of Computing Machinery
AHP: Analytical Hierarchical Process
IEEE: Institute of Electrical and Electronics Engineers
NCBO: National Center for Biomedical Ontologies
NLG: Natural language generation
OWL2: Web Ontology Language, version 2
RDF: Resource Description Framework
SUS: System Usability Scale
URI: Uniform Resource Identifier
